# Слезный аппарат как орган риска при проведении радионуклидной терапии

**DOI:** 10.14341/probl13163

**Published:** 2024-02-27

**Authors:** М. С. Шеремета, А. А. Трухин, В. Д. Ярцев, Д. В. Юдаков, М. О. Корчагина, С. А. Годжаева

**Affiliations:** Национальный медицинский исследовательский центр эндокринологии; Национальный медицинский исследовательский центр эндокринологии; ФГБНУ «НИИ глазных болезней им. М.М. Краснова»; Национальный медицинский исследовательский центр эндокринологии; Национальный медицинский исследовательский центр эндокринологии; ФГБНУ «НИИ глазных болезней им. М.М. Краснова»; Первый московский государственный медицинский университет имени И.М. Сеченова

**Keywords:** онкоэндокринология, офтальмология, 131-йод, 177-лютеций, радионуклидная терапия, вторичная облитерация слезоотводящих путей, слезные железы.

## Abstract

В рамках статьи авторы анализировали доступные сведения о поражении слезного аппарата при проведении радио­нуклидной терапии. Отдельно рассмотрены описания поражений системы слезопродукции, главной и добавочных слезных желез, а также слезоотведения. Обнаружено, что поражение слезного аппарата характерно для терапии 131I по поводу рака щитовидной железы, а также для радиолигандной терапии с применением анти-PSMA-антител, меченных 177Lu и 225Ac. При терапии 131I возможно формирование вторичной облитерации слезоотводящих путей, а при применении 177Lu-PSMA и 225Ac-PSMA возможно поражение слезной железы с формированием клинически выраженного «синдрома сухого глаза». Патогенез таких поражений связан с накоплением радиоизотопа в тканях слезного аппарата, накопление при проведении терапии 131I реализуется в связи с экспрессией натрий-йодного симпортера в носослезном протоке, а при проведении терапии 177Lu-PSMA и 225Ac-PSMA радиобиологический эффект реализуется в связи с экспрессией PSMA тканью слезной железы. Анализ доступных источников показал, что на сегодняшний день отсутствуют результаты систематических исследований, посвященных проблеме, имеется дефицит знаний, касающихся индивидуальных рисков развития этих осложнений, не разработаны способы их профилактики, имеющие доказанную эффективность, а применяющиеся методы лечения имеют относительно низкую эффективность. Авторы приходят к заключению, что усиление междисциплинарного взаимодействия, а также организация методологически выверенных и корректных исследований может способствовать решению задач, связанных с изучением рассматриваемых осложнений.

## Abstract (en)

Within the framework of the article, the authors analyzed the available information about the damage to the lacrimal apparatus during radionuclide therapy. In focus of article lesions of the lacrimal production system, the main and accessory lacrimal glands, as well as lacrimal drainage are considered. It was found that damage to the lacrimal apparatus is characteristic of 131I therapy for thyroid cancer, as well as for radioligand therapy using anti-PSMA antibodies labeled with 177Lu and 225Ac. 177Lu-PSMA and 225Ac-PSMA may damage the lacrimal gland with the formation of a clinically pronounced "dry eye syndrome". The pathogenesis of such lesions is associated with the accumulation of a radioisotope in the tissues of the lacrimal apparatus, while during therapy with 131I, accumulation is realized due to the expression of the sodium-iodine symporter in the nasolacrimal duct, and during therapy with 177Lu-PSMA and 225Ac-PSMA, the radiobiological effect is realized in connection with the expression PSMA by lacrimal tissue. An analysis of the available sources showed that to date there are no results of systematic studies on the problem, there is a lack of knowledge regarding the individual risks of developing these complications, methods for their prevention that have proven effectiveness have not been developed, and the treatment methods used, having relatively low efficiency, are not specialized. The authors concluded that the strengthening of interdisciplinary interaction, as well as the organization verification methodology and correct studies, can contribute to solving problems related to the study of the complications under consideration.

Слезный аппарат представляет собой комплекс, функционально состоящий из двух частей: системы слезопродукции и системы слезоотведения. Слезная жидкость образуется главной слезной железой, а также множеством добавочных, находящихся в конъюнктиве и веках. Система слезоотведения анатомически состоит из расположенных на верхнем и нижнем веках слезных точек, продолжающихся слезными канальцами, слезным мешком и носослезным протоком, который открывается в нижний носовой ход. Функционирование слезной системы состоит в непрерывном образовании слезной жидкости, которая омывает глазную поверхность и по слезоотводящим путям эвакуируется в носовую полость.

При нарушении слезопродукции возникает дефицит слезной жидкости, вследствие чего формируется «синдром сухого глаза», при недостаточности слезоотведения возникает слезотечение, сопровождающееся развитием воспалительных изменений на глазной поверхности и в просвете слезоотводящих путей.

Радионуклидная терапия может сопровождаться присутствием радиоактивного изотопа в слезной железе и слезоотводящих путях, т.к. слезная жидкость представляет собой фильтрат плазмы крови [1]. Слезный аппарат — это зона повышенного накопления радиоизотопа при проведении радионуклидной терапии и диагностики радиофармацевтическими лекарственными препаратами (РФЛП) на основе 131I, 177Lu, 225Ac, 68Ga, 99mTc и др. [2–4]. С клинической точки зрения, после однократного курса радиойодтерапии 131I в 9% случаев развивается вторичная облитерация слезоотводящих путей, а в случае увеличения суммарной активности при повторных курсах вероятность развития вторичной облитерации слезоотводящих путей возрастает до 25% [5]. Радионуклидная терапия, проводимая по поводу метастазов рака предстательной железы PSMA лигандами, меченными 177Lu, 225Ac, 68Ga, сопровождается интенсивным накоплением РФЛП в слезных железах, что объясняет риск развития постлучевых осложнений в виде нарушения слезопродукции [4][6][7]. ТЕРАПИЯ 131-ЙОДОМ (131I) В литературе представлено описание случаев развития осложнений со стороны слезной системы у пациентов, получавших терапию радиоактивным йодом. Описаны случаи развития ксеротических изменений («синдрома сухого глаза») со стороны глазного яблока, связанные с поражением слезных желез [8–11], а также случаи развития осложнений со стороны носослезного протока, когда формируется облитерация путей слезоотведения [5][12–16]. Радиоактивный изотоп йода (131I) накапливается в тиреоцитах, что позволяет использовать его в качестве диагностического и терапевтического агента у пациентов с заболеваниями щитовидной железы. Транспорт 131I осуществляется с помощью белка-переносчика ионов йода — натрий-йодного симпортера (НЙС). НЙС — это гликопротеин цитоплазматической мембраны, который реализует активный транспорт йода в щитовидной железе и других тканях, таких как слюнные железы, желудок, лактирующие молочные железы, тонкий кишечник и плацента [17]. В щитовидной железе НЙС-опосредованное поглощение йода играет ключевую роль в биосинтезе гормонов щитовидной железы, а при проведении терапии радиоактивным йодом при помощи НЙC реализуется концентрирование 131I в щитовидной железе и развивается местный радиобиологический эффект. Однако поглощение экстратиреоидными тканями 131I способствует возникновению нежелательных побочных эффектов радиойодтерапии. Известно, что тиреотропный гормон (ТТГ) стимулирует поглощение 131I через активацию транскрипции SLC5A5 — гена, кодирующего белок НЙС, поэтому для повышения эффективности радиойодтерапии перед ее проведением увеличивают концентрацию ТТГ плазмы крови. Вместе с тем рецепторы ТТГ обнаруживаются не только в тканях щитовидной железы, но и экстратиреоидно, что обуславливает захват 131I тканями, осуществляющими значимую экспрессию НЙС. Таким образом, для проведения терапии радиоактивным йодом теоретически необходимо максимально увеличить экспрессию НЙС тканями щитовидной железы для реализации максимального местного радиобиологического эффекта, одновременно снизив до минимума экспрессию НЙС вне щитовидной железы для снижения вероятности развития экстратиреоидных осложнений. Однако на сегодняшний день способов, позволяющих повысить восприимчивость щитовидной железы к 131I при одновременном ее снижении вне щитовидной железы, не разработано. Поражение слезной железы Проведенные ретроспективные исследования показали, что «синдром сухого глаза», возникающий после радиойодтерапии, связан с поражением не только главной слезной железы, но и добавочных желез; при этом происходит нарушение выработки как водного, так и липидного компонентов слезной пленки. Кроме того, снижение уровня слезопродукции происходит даже в случае терапии малыми активностями [9–11][18][19]. Клиническим проявлением поражения слезных желез и нарушения слезопродукции является развитие сухого кератоконъюнктивита. Стоит отметить, что почти в четверти наблюдаемых случаев изменения имеют транзиторный характер и не требуют какой-либо специальной коррекции [11][20][21]. Авторы, проводившие исследования, которые касались патоморфологических изменений слезной железы после воздействия 131I, пришли к заключению, что в основе механизма поражения лежит оксидативный стресс, индуцированный ионизирующим излучением [22]. Согласно распространенному мнению, ионизирующее излучение приводит к развитию реакций, вследствие которых образуются свободные радикалы, которые взаимодействуют с внутриклеточными мембранами, нарушая их целостность [23][24]. Полученные в ходе патоморфологических исследований на животной модели результаты показали, что непосредственно после воздействия 131I в ткани слезной железы возникает выраженный воспалительный процесс, который сопровождается метаплазией эпителиальных клеток, а также апоптотическими изменениями на фоне индуцированного радиационным воздействием фиброза [8][22][25]. Проведенные исследования не выявили корреляций между частотой и/или степенью поражения слезной железы и введенной активностью 131I [9][11]. К настоящему моменту не разработаны какие-либо схемы профилактики осложнений радиойодтерапии со стороны слезной железы, которые имели бы доказанную клиническую эффективность. Ведутся экспериментальные исследования, в рамках которых изучают возможности антиоксидантной защиты медикаментозными средствами-антиоксидантами: витаминами D и E, коэнзимом Q10, препаратами на основе цинка, ликопина и ресвератрола [8][22][25–27]. Нами не найдено клинических рекомендаций по специфическому лечению данного осложнения. Облитерация слезоотводящих путей Несмотря на то что физиологическая роль захвата йода слизистой оболочкой носослезного протока на сегодняшний день неясна, иммуногистохимическими исследованиями подтверждена экспрессия НЙС в дистальных отделах носослезного протока [2]. При помощи лучевых исследований подтверждено наличие в некоторых случаях фиксации в носослезном протоке радиоактивного йода после проведения радиойодтерапии [28–30]. Несмотря на то что максимальный захват 131I, очевидно, происходит в первые часы после введения радиологической активности, клинические проявления в виде развития дакриостеноза и непроходимости слезоотводящих путей реализуются через несколько месяцев [5][12][13][15]. Вероятность развития клинически значимого дакриостеноза возрастает пропорционально величине введенной активности [15][28]. Частота развития нарушения слезоотведения, по различным оценкам, находится в диапазоне от 5 до 18% случаев при однократном курсе радиойодтерапии [14][15][31][32]. Патоморфологический анализ изменений, происходящих в области устья носослезного протока у пациентов с развившейся непроходимостью слезоотводящих путей после терапии радиойодом, показал, что они имеют типичный характер. Так, обнаружена десквамация эпителия носослезного протока и нарушение морфологии слизистых желез на фоне умеренного фиброза [33]. Специфических методов профилактики этого осложнения, а также патогенетически обоснованных методов лечения не предложено. Большинство авторов сходятся во мнении, что целесообразно назначать глазные лубриканты, слезозамещающие и противовоспалительные капли [12][14][16]. К настоящему времени отсутствуют сведения о результатах достоверных с позиций доказательной медицины исследований, показавших эффективность каких-либо способов профилактики накопления 131I в слезоотводящих путях, а также реализации радиобиологического эффекта такого накопления. Авторы посвященных этому вопросу исследований полагают, что теоретически положительный эффект может быть достигнут при применении амифостина, а также при конкурентном ингибировании НЙС носослезного протока перхлоратом натрия. Вместе с тем на сегодняшний день результатов экспериментальных или клинических исследований по этому вопросу представлено не было [12][16]. В случае возникновения клинически значимой непроходимости слезоотводящих путей прибегают к классическим оперативным вмешательствам [12][13][15][28][31][32], хотя известен по крайней мере один случай эффективной реканализации слезоотводящих путей, выполненной под трансканаликулярным эндоскопическим контролем [34]. ТЕРАПИЯ 177-ЛЮТЕЦИЕМ (177LU), 225-АКТИНИЕМ (225АС) В клинической практике 177Lu-PSMA, 225Ac-PSMA используют у пациентов, страдающих раком предстательной железы, в рамках радиолигандной терапии [6][35]. Эпителий предстательной железы экспрессирует белок PSMA (prostate specific membrane antigen — простатспецифический мембранный антиген); аналогичная экспрессия характерна для клеток некоторых видов рака предстательной железы. Проведенные исследования показали, что PSMA обнаруживается также вне предстательной железы, в частности в тканях почки, слюнных и слезных желез, однако концентрация PSMA в опухолях, как правило, значительно превышает таковую в неизмененных тканях. При проведении радиолигандной терапии системно вводят анти-PSMA антитело, меченное изотопами 177Lu и 225Ac, которые являются источниками β- и α-излучения соответственно. Механизм действия радиолигандной терапии состоит в том, что при возникновении реакции антиген-антитело происходит концентрирование радиоизотопа в месте экспрессии антигена, в данном случае PSMA. При этом эффект развивается и вне предстательной железы при условии значимой экспрессии PSMA. Поражение слезной железы В ткани слезной железы активно экспрессируется PSMA, что приводит к избыточному накоплению 177Lu и 225Ac при проведении радиолигандной терапии [4][36][37]. Физиологическая роль экспрессии PSMA слезной железой до сегодняшнего дня остается неясной. В то же время показано, что при некоторых заболеваниях, связанных с непосредственным поражением слезной железы, в частности при синдроме Sjögren, отмечено снижение экспрессии этого белка, а отклонение его значений от нормальных может рассматриваться как диагностический признак некоторых дегенеративных состояний слезной железы [38]. По различным данным, «синдром сухого глаза» развивается у 3–29% пациентов, перенесших терапию 177Lu-PSMA [39][40]. Сведений о вероятности развития клинически выраженного «синдрома сухого глаза» при терапии 225Ac-PSMA к настоящему времени не представлено.

## ЗАКЛЮЧЕНИЕ

Таким образом, слезный аппарат представляет собой орган риска при проведении радионуклидной терапии. Индуцированные нарушения могут касаться как системы слезопродукции, так и системы слезоотведения.

Наиболее актуальными задачами в контексте рассматриваемой проблемы нам представляется: исследование индивидуальных факторов риска развития нарушения слезопродукции и слезоотведения у пациентов, получающих соответствующее лечение, а также выявление взаимосвязи между протоколом радионуклидной терапии, клиническими особенностями, а также дакриологическим статусом пациента в момент проведения терапии; разработка персонализированных способов профилактики осложнений.

На сегодняшний день способы устранения осложнений не отличаются от способов коррекции первичных нарушений слезопродукции и слезоотведения, при этом их относительная эффективность ниже у пациентов с радиологически индуцированными нарушениями. Вместе с тем наличие сведений об особенностях патоморфологических изменений объясняет необходимость разработки специальных хирургических вмешательств у этой группы больных, методов профилактики и лечения осложнений со стороны слезного аппарата.

Проведение совместных многоцентровых исследований, не содержащих методологических погрешностей, позволит расширить понимание патогенеза рассматриваемых осложнений, а значит, станет возможна разработка клинически эффективных способов профилактики поражения слезного аппарата при радионуклидной терапии.

## ДОПОЛНИТЕЛЬНАЯ ИНФОРМАЦИЯ

Источник финансирования. Материал подготовлен в рамках реализации государственного задания Министерства здравоохранения Российской Федерации, регистрационный номер ЕГИСУ НИОКТР 123021000041-6 «Исследование фармакобезопасности тераностических радиофармацевтических лекарственных препаратов с использованием гибридной молекулярной визуализации в диагностике и лечении эндокринных и онкологических заболеваний в детской и взрослой возрастных группах»; при подготовке рукописи авторы сохранили независимость мнений.

Конфликт интересов. Авторы декларируют отсутствие явных и потенциальных конфликтов интересов, связанных с содержанием настоящей статьи.

Участие авторов. Трухин А.А. — существенный вклад в концепцию исследования, а также в получение и анализ данных, написание статьи и внесение в рукопись существенной (важной) правки с целью повышения научной ценности статьи; Ярцев В.Д. — существенный вклад в концепцию исследования, а также в получение и анализ данных, написание статьи и внесение в рукопись существенной (важной) правки с целью повышения научной ценности статьи; Шеремета М.С. — существенный вклад в концепцию исследования, а также в получение и анализ данных, написание статьи и внесение в рукопись существенной (важной) правки с целью повышения научной ценности статьи; Юдаков Д.В. — существенный вклад в концепцию исследования, а также в получение и анализ данных, написание статьи и внесение в рукопись существенной (важной) правки с целью повышения научной ценности статьи; Корчагина М.О. — существенный вклад в концепцию исследования, а также в получение и анализ данных, написание статьи и внесение в рукопись существенной (важной) правки с целью повышения научной ценности статьи; Годжаева С.А. — существенный вклад в концепцию исследования, а также в получение и анализ данных, написание статьи и внесение в рукопись существенной (важной) правки с целью повышения научной ценности статьи.

Все авторы одобрили финальную версию статьи перед публикацией, выразили согласие нести ответственность за все аспекты работы, подразумевающую надлежащее изучение и решение вопросов, связанных с точностью или добросовестностью любой ее части.

Благодарности. Авторы выражают признательность руководству ФГБУ «НМИЦ эндокринологии» Минздрава России и ФГБНУ «НИИ глазных болезней им. М.М. Краснова» за оказанную юридическую поддержку в объединении междисциплинарных усилий для исследования фармакобезопасности при применении радиофармацевтических лекарственных препаратов.
